# Whole-Genome Sequence of Listeria monocytogenes Strains from Clinical and Environmental Samples from Varanasi, India

**DOI:** 10.1128/genomeA.01496-14

**Published:** 2015-02-05

**Authors:** Dharmendra K. Soni, Krishna M. Singh, Arpita Ghosh, Surendra K. Chikara, Chaitanya G. Joshi, Suresh K. Dubey

**Affiliations:** a Centre of Advanced Study in Botany, Banaras Hindu University, Varanasi, India; b Xcelris Genomics, Xcelris Labs Ltd., Old Prechandnagar, Opp Satyagrah Chawani, Bodakdev, Ahmedabad, Gujarat, India; c Department of Animal Biotechnology, Anand Agriculture University, Anand, Gujrat, India

## Abstract

We present here the whole-genome sequences of Listeria monocytogenes from Ganges River water, agricultural soil, and human clinical samples from Varanasi, India, which will be used for a comparative analysis.

## GENOME ANNOUNCEMENT

Listeria monocytogenes is the Gram-positive pathogenic bacterium that causes listeriosis and results in high mortality (20 to 30%) and hospitalization rates (1, 2). L. monocytogenes encompasses a variety of strains that vary in their pathogenic potential in hosts (3, 4). L. monocytogenes strains group into 13 serotypes, of which 1/2a, 1/2b, and 4b represent the majority of the pathogenic strains (5, 6). To obtain a better understating of the molecular mechanisms of L. monocytogenes pathogenicity with special reference to their different ecological niche, the genome sequences of the isolates recovered from Ganges River water, agricultural soil, and human clinical samples (placenta bit) were analyzed.

Genomic DNA was extracted using the QIAamp DNA minikit (Qiagen, Milan, Italy), according to the manufacturer’s protocol. Whole-genome shotgun sequencing was performed using the 318 Chip and 300-bp chemistry Ion Torrent PGM platform, per the manufacturer’s instructions. The reads were quality filtered using Prinseq-lite-0.20.3. *De novo* assembly was carried out using Newbler version 2.6. The gene annotation and screening for RNAs were performed by submitting the sequences to the Rapid Annotations using Subsystems Technology (RAST) server (7). The resulting assemblies generated 13, 19, and 15 contigs representing the genomes of L. monocytogenes BHU1 (Ganges River water), L. monocytogenes BHU2 (agriculture soil), and L. monocytogenes BHU3 (human placenta bit), respectively, with an average sequencing coverage of ~89× for all three samples. The estimated genome size is 2.9 Mb, and G+C contents of 39%, 38%, and 38% were evident for the L. monocytogenes strains BHU1, BHU2, and BHU3, respectively. The annotation pattern suggested the genomes to contain 3,030, 3,011, and 3,005 protein-coding genes (CDSs) for L. monocytogenes BHU1, BHU2, and BHU3, respectively, which includes the hypothetical and RNA genes.

The genome analysis showed all the isolates to possess a relatively high number of CDSs involved in carbohydrate and amino acid metabolism, as well as virulence-defense mechanisms. More significantly, the availability of these L. monocytogenes genome sequences offers the opportunity to conduct further comparative genomics studies between pathogenic and nonpathogenic isolates and their listeriosis potential in India. This will also facilitate the development of newer comprehensive genotype assays for L. monocytogenes.

### Nucleotide sequence accession numbers.

The whole-genome sequences described here have been deposited in DDBJ/EMBL/GenBank under the accession numbers JUKE00000000, JUKF00000000, and JUKG00000000 for L. monocytogenes BHU1, L. monocytogenes BHU2, and L. monocytogenes BHU3, respectively.
